# The Impact of Hemodialysis on Spatio-Temporal Characteristics of Gait and Role of Exercise: A Systematic Review

**DOI:** 10.3390/healthcare5040092

**Published:** 2017-12-05

**Authors:** Anuradha Sawant, Tom Overend

**Affiliations:** 1 London Health Sciences Center, London, ON N6G 5A5, Canada; 2 Department of Physiotherapy, Western University, London, ON N6G 1H1, Canada; toverend@uwo.ca

**Keywords:** gait speed, 6 min walk test, timed up-and-go test, hemodialysis, end-stage kidney disease

## Abstract

*Background:* People with end-stage kidney disease (ESKD) on hemodialysis (HD) commonly have functional impairments. The purpose of this systematic review was to evaluate the effect of HD on spatio-temporal characteristics of gait, and effect of exercise on these parameters. *Methods:* Electronic databases were searched to identify relevant citations. Extracted data was computed using a random effects model for means (Hedges’ and 95% confidence interval (CI). *Results:* 27 studies met inclusion criteria. Mean values: gait speed (GS)—1.0 m/s (CI: 0.9–1.1 m/s; 16 studies), fast walking speed (FWS)—1.5 m/s (CI: 1.3–1.6 m/s; 7 studies), timed get-up & go test (TUG) —6.8 s (CI: 6.1–7.5 s; 2 studies), walk tests (WT) 193.0 s (CI: 116.0–270.0; 5 studies), 6 min-walk-test (6MWT)—386.6 m (CI: 243.2–530.0 m; 11 studies). 4 studies compared participants on HD with normal controls and 10 studies evaluated the effect of nutrition/exercise. *Conclusions:* Compared to age-matched populations, people with ESKD/HD had significantly slower GS and reduced walk distances; with intervention, the change in the distance walked was significant. Further research is required to evaluate the effect of HD on gait parameters, and the type of exercise/nutrition that will lead to meaningful changes.

## 1. Introduction

Chronic kidney disease is the progressive failure of renal function over a period of years. In its end-stage, renal replacement therapies (RRT) such as hemodialysis (HD), peritoneal dialysis (PD), or kidney transplants are required to supplement the metabolic homeostatic functions of the kidney. The aging of the Canadian population is reflected in the demographic profile of new end-stage kidney diseases (ESKD) patients: 53% of those who initiated RRT in 2010 were aged 65 years and older, compared to 39% in 1991 [1].

People with ESKD on HD experience multiple catabolic processes, including loss of albumin and amino acids during dialysis, metabolic derangements, and changes in skeletal muscle associated with conditions of muscle disuse [2]. These changes result in muscle atrophy (loss of lean muscle mass). The presence of neurogenic (muscle atrophy or loss associated with nerve disorder), myogenic (damage intrinsic to the muscle), and mixed (neurogenic and myogenic) changes intrinsic to the skeletal muscle in people with ESKD on HD [3] may further compromise the integrity of the motor-unit complex and contribute to muscle atrophy [4]. As a result, such changes can lead to overall decreases in gait and mobility [5].

People with ESKD on HD are also known to have renal osteo-dystrophy [6] predisposing them to increased risk for falls [7,8] and long bone fractures [9]. This incidence of falls in people on HD is higher than their non-uremic community-dwelling counterparts; for the community-dwelling older adults, the fall rate ranges from 0.32 to 0.70 fall/person-year [10,11] whereas the same amongst people requiring HD ranges from 1.18 [7] to 1.6 [8] falls per person-year. Since falls commonly predict morbidity, mortality and perhaps need for institutional care, it is important to initiate appropriate interventions in a timely manner to prevent falls and related consequences [11].

The deleterious consequences of falls are not restricted to wounds, fractures, hospitalization, or death. Post-fall anxiety syndrome and fear of falling again result in a loss of self-confidence and self-restriction of activity creating a vicious circle leading to reduced exercise and muscle mass. Falls also are a leading cause of admission to nursing homes. Finally, the cost inherent to falls is substantial: they account for 6% of all medical expenditures in non-uremic patients 65 years or older [10].

Walking speed (WS) or gait speed (GS) is a well-recognized prognostic factor in the geriatric population. It provides information on patient outcomes and hospitalization risk [12]. It has been considered a reliable and sensitive outcome to measure functional abilities, and ability to predict future health status and quality of life. Fritz and Lusardi [13] argue that GS can be used as a functional “vital sign” to help determine risk for falls in people on HD [14], and outcomes such as functional status [15,16], discharge location [17], and the need for rehabilitation in geriatric population [18]. The clinical findings of impaired GS may alert the health care professionals to initiate appropriate exercise interventions and/or introduce suitable gait-aids for that individual. Therefore, it is essential to understand the impact of HD on spatial (distance walked) or temporal (gait speed) characteristics of gait. The primary objective of this study was to determine the effects of HD on spatio-temporal gait parameters. The secondary objective was to review the effect of exercises on these parameters within the cluster of studies selected for this review.

## 2. Materials and Methods

### 2.1. Literature Search

Electronic databases (PubMed, Medline, Embase, EBSCO, and Scopus) were searched from their inception until May 2017, using different strategies that encompass the wide range of gait parameters that may be affected by HD. The following keywords were searched as MeSH or “mapped terms” and as text words: (chronic OR end-stage) AND (kidney OR renal) AND (disease OR failure) AND (dialysis OR hemodialysis OR haemodialysis) AND (walk OR walking OR ambulation OR ambulatory OR ambulate OR gait) and (speed OR velocity).

### 2.2. Inclusion Criteria

The inclusion criteria were as follows: (a) the paper was published in the English language; (b) participants on HD were adults; (c) one of the outcome measures measured spatial or temporal parameters of gait (d) full text articles was available.

### 2.3. Exclusion Criteria

The exclusion criteria were the following: (a) study participants on peritoneal dialysis or any other form of renal replacement therapy other than HD; (b) animal studies or trials; (c) case studies or literature reviews.

### 2.4. Outcome Measures 

Outcome measures such as GS, timed up-and-go test (TUG) [19], six minute walk test (6MWT) [20], and the intermittent shuttle walk test (ISWT) [21], were considered as appropriate outcome measures for inclusion in this review. All gait speed measurements that were reported in formats other than meters per second (m/s) were adjusted to meet this standardized unit of GS as m/s.

### 2.5. Data Collection and Analysis

Following the primary search, the primary authors independently reviewed the titles, abstracts and full texts to establish the inclusion of the study for this review. Discussions in case of discrepancy were adequate for resolution. All articles were included by consensus.

Required data for calculations of cumulative means and the 95% confidence interval (CI) around the mean were extracted using a standardized form, from the included studies by one author and reviewed by the second author for accuracy. In studies with experimental designs baseline values of the outcome measures in various groups was included for estimation of effect of HD.

### 2.6. Statistical Analyses

A statistical package, Comprehensive Meta-Analysis (Version 2.2.064, Biostat, Englewood, NJ, USA) software for meta-analysis of binary, continuous and diagnostic data, was used for computation of the cumulative means. The confidence interval at a 95% confidence limit was constructed around the point estimate of the cumulative mean.

The results reported were calculations using the random effects model to account for methodological differences amongst studies. The significance level for all statistical tests evaluating the effect of exercises or comparison with normal was set at *p* < 0.05.

## 3. Results

**Figure 1:**
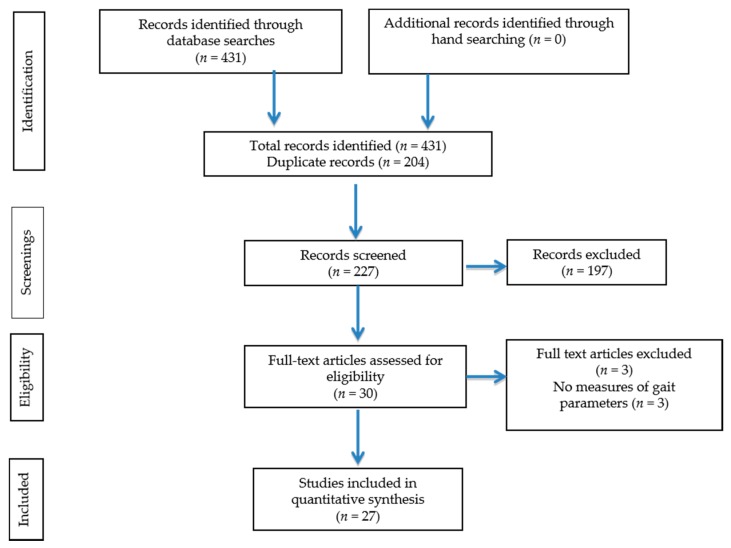
Flow Chart of Study Selection for the systematic review.

The initial search yielded 431 citations. Of these 27 [5,22,23,24,25,26,27,28,29,30,31,32,33,34,35,36,37,38,39,40,41,42,43,44,45,46,47] studies met inclusion criteria (Figure 1). Characteristics of the studies included are presented in Table 1. Sixteen [5,22,23,25,27,31,32,33,34,35,36,37,39,40,42,45] of the 27 studies published a single point data, four studies [28,29,30,34] utilized pre-post design to evaluate the effect of exercises and seven studies [24,26,38,41,44,46,47] used a controlled trial design to evaluate effect of either nutritional or exercise intervention in this population. The baseline values of the participants in these studies were utilized for analysis evaluating the effect of HD on spatio-temporal gait parameters.

### 3.1. Effect of Hemodialysis on Spatiotemoporal Gait Parameters

Gait speed (GS): A total of 16 studies [5,23,25,27,29,32,33,34,35,36,37,39,44,45,46,47] reported GS in 23 sub-groups of participants (Table 2). The mean GS was 1.01 m/s (95% CI: 0.95–1.07 m/s).

Fast walking speed (FWS): A total of 7 studies [22,23,29,39,42,44,46] reported FWS in 8 sub-groups of participants (Table 3). The mean FWS was 1.4 m/s (95% CI 1.3–1.6 m/s).

Timed up and go test (TUG): A total of 2 [38,47] studies reported TUG in 6 sub-groups of participants (Table 4). The mean value of TUG was 6.8 s (95% CI 6.1–7.5 s).

6 min walk test (6MWT): A total of 10 studies [24,25,26,27,29,30,38,41,42,44] reported 6MWT in 16 sub-groups of participants. The mean 6MWT distance in this group was 411.6 (95% CI: 377.0–446.1 m) (Table 5).

Walk tests: A total of 5 studies reported walk tests presented as time required in seconds for the completion of tests (Table 6). Bulckaen et al. [24] reported treadmill walk tests, Jeong et al. [32] reported intermittent shuttle walk test (ISWT), Lane et al. [40] and Tomayako et al. [47] reported shuttle walk tests (SWT), and Mercer et al. [43] reported 50 m walk tests. The data extracted from these studies was grouped together to estimate the effect of HD on walk timings. The mean time taken to complete these tests was 193.5 s (95% CI 116.3–270.7 s).

Outcome measures “Number of steps taken per day” [24] and GAITRite mat data [45] was reported by only one study each. Hence, these outcome measures were not included in the meta-analysis. Findings from these studies are summarized in Table 1.

### 3.2. Comparison with Normal Controls

Four studies [5,23,35,45] presented comparison of GS with normal control participants or established normative values (Table 7). For the GS, participants on HD were slower by 1.26 m/s (95% CI 0.76–1.76 m/s; *p* < 0.001) when compared to age-matched normal controls with no kidney disease. Table 8 presents the analysis from the GAITRite investigation by Shin et al. [45].

### 3.3. Effect of Exercise or Nutritional Intervention on Spatiotemoporal Gait Parameters

**Figure 2:**
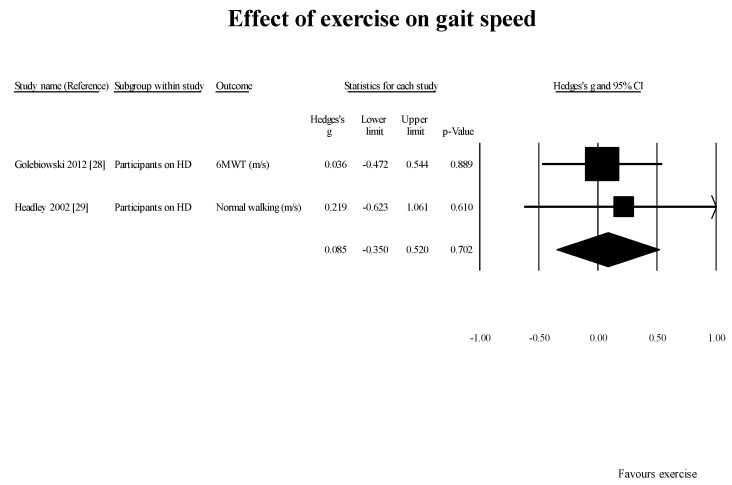
Results of effect of exercise on gait speed in studies using pre-post design. 6MWT: six min walk test; HD: hemodialysis; m/s: meters per second.

**Figure 3:**
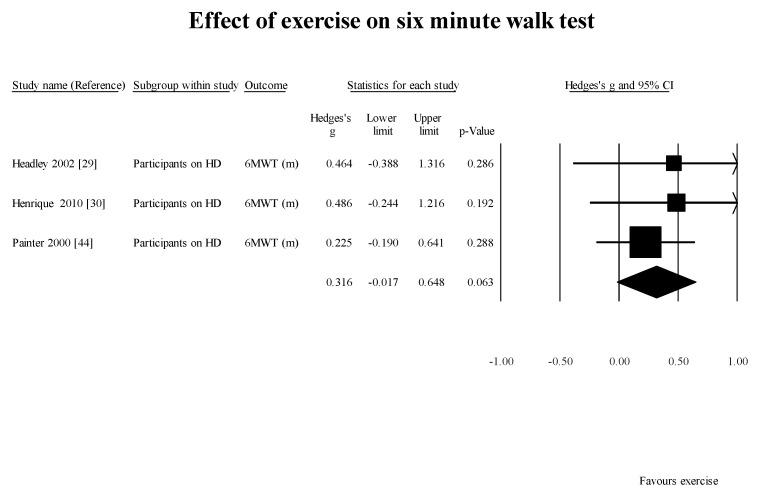
Results of effect of exercise on 6MWT in studies using pre-post design. 6MWT: six minute walk test; HD: hemodialysis.

**Figure 4:**
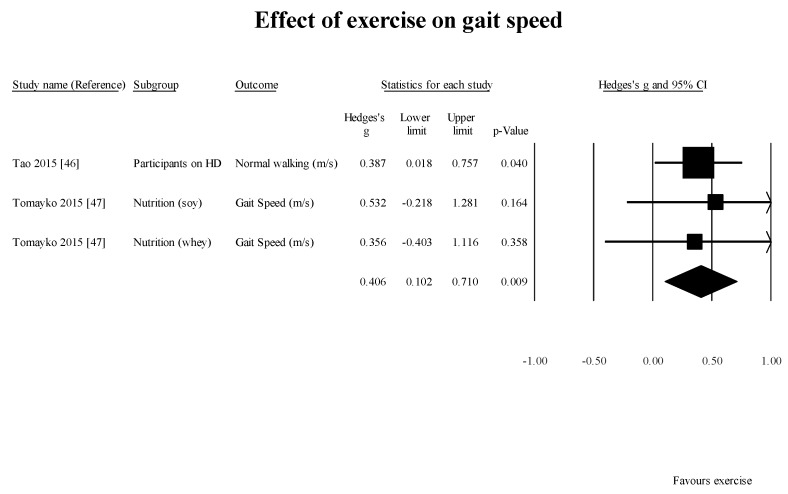
Results of effect of exercise on gait speed in studies using controlled study design.

**Figure 5:**
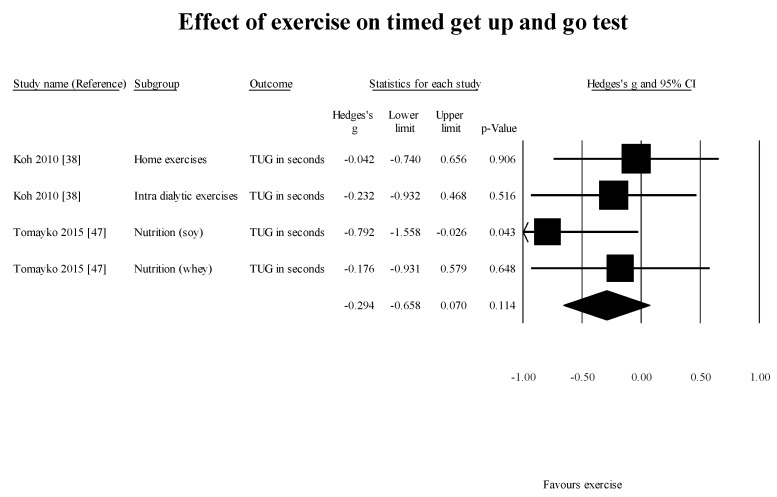
Results of effect of exercise on TUG in studies using controlled study design. TUG: timed get-up and go test.

**Figure 6:**
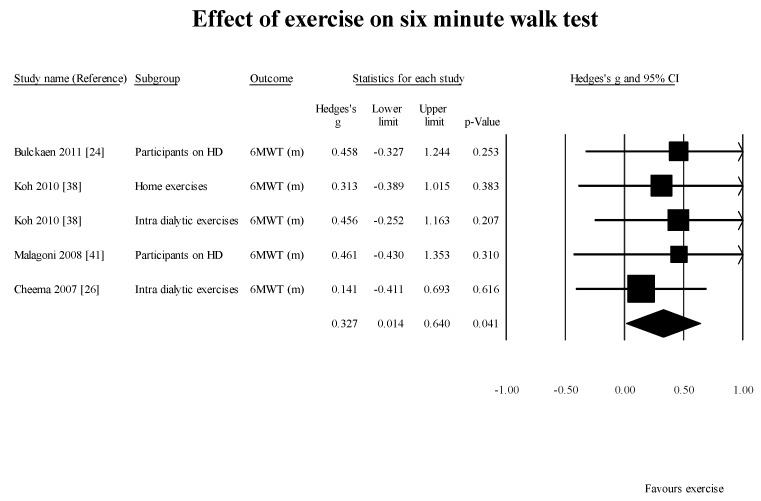
Effect of exercises on six minute walk test. 6MWT: six minute walk test; HD: hemodialysis; m: meter.

Studies using pre- and post-design for intervention: Three studies [29,30,44] within the cluster of studies included in this review utilized pre- and post-intervention study design to evaluate the effect of exercises in three subgroups of participants. The mean changes were as follows:

GS [28,29] (2 studies, 2 subgroups): 0.08 m/s (95% CI: −0.35–0.52; *p* = 0.7) (Figure 2);

FWS [29] (1 study, 1 group): 0.92 m/s (95% CI: 0.0–1.85; *p* = 0.05); 6MWT [28,29,44] (3 studies 3 subgroups): 0.32 m/s (95% CI: −0.02–0.65; *p* = 0.0.06) (Figure 3);

Studies using intervention and control design for intervention: Six studies within the cluster of studies included in this review utilized controlled experimental study design to evaluate the effect of exercises/nutrition in eight subgroups of participants. The mean changes were as follows: 

GS [46,47] (2 studies, 3 subgroups): 0.8 m/s (95% CI: 0.10–0.71; *p* = 0.01) (Figure 4);

FWS [46] (1 study, 1 group): 0.34 m/s (95% CI: −0.02–0.72; *p* = 0.06); TUG [38,47] (2 study, 4 group): −0.29 s (95% CI: −0.66–0.07; *p* = 0.11) (Figure 5);

6MWT [24,26,38,41] (4 studies, 5 groups): 0.33 m (95% CI: 0.01–0.64; *p* = 0.04) (Figure 6);

Walk tests [24,43,47] (3 studies 4 subgroups): 0.91 s (95% CI: 0.49–1.32; *p* < 0.001).

## 4. Discussion

The results of this systematic review confirm the earlier findings of reduced GS (normal walking) and distance walked in six minutes by participants on HD in the literature. The mean dialysis vintage of the participants included in the studies reporting dialysis vintage was 53 months. This indicates that within a period of 53 months, people on HD have a significantly slower gait speed when compared to an age-matched population without ESKD. One of the studies [47] selected in this review did not report the dialysis vintage of the participants in the study. According to Roshanravan et al. [48], GS measured with a 4-m walk test in participants with CKD stages 2 to 4 was about 30% lower than predicted. Hence, at this time it is unclear whether the participants who progressed to requiring HD continued to decline after the treatment was started. Further longitudinal studies are required to elucidate the effect of HD on GS as a function of time.

We evaluated the effect of whole body fluid loss following HD on tibialis anterior (TA) strength and water content [49]. Overall, a significant reduction (*p* < 0.05) in peak strength by 1.54 Nm (95% CI: 0.05, 3.02), and extra cellular fluid (ECF) (measured using transverse relaxation times on magnetic resonance spectroscopy (T_2_) shortened by 2.38 ms; 95% CI: 1.04, 3.71) of TA were observed between before- and after-HD measurements. Based on these findings, we recommended that deteriorating muscle functions such as gait and mobility should be added as a symptom of chronic dehydration, requiring further assessment of dry weight of a HD patient. This is important, as reduction in muscle strength has been associated with whole-body fluid loss [50], and Edwards et al. [51] suggest that a weaker muscle, unable to meet functional demands such as standing or walking made upon it, leads to further muscle weakness.

Although we set out to evaluate the effect of HD on GS, we did not find any longitudinal study reporting the time frame following HD when measurable reductions in GS and/or mobility occur. However, we recommend that gait assessments be conducted at three-month intervals as Johansen and colleagues [28] have demonstrated a decrement in the Human Activity Profile (HAP) Questionnaire (measure of self reported physical activity) adjusted activity score (HAP–AAS) scores in HD participants at a follow-up data collection after three months.

Two [46,47] of the six case-control studies evaluating the effect of exercise established a significant increment in GS. These findings are in alignment with what has been reported in the literature [52,53]. However, adherence and uptake of these exercises is limited [54]. Hence, it is important to identify people on HD with deteriorating GS or self-reported physical function. These findings can lead to initiating multi-disciplinary intervention to optimize patient’s functional abilities, reduce the risk for falls and related co-morbidities.

Another factor requiring further investigation is whether supervised exercise is superior to home-based exercise for improving GS among these patients. All the case-control studies included in this review did not conclusively determine if home-based exercise program or supervised exercise programs are superior. Tomayako et al. [47] indicated that a supervised exercise program resulted in improved gait speed, whereas Koh et al. [38] suggested that home-based exercises were as effective as supervised exercise programs.

Donat and Özcan [55] compared the effectiveness of supervised group exercise and unsupervised home exercise programs on parameters related to risk of falls among older adults. Both groups showed significant improvements in balance, but the supervised group also showed significant improvement in strength and proprioception, which are important, factors in overall postural control. There are several reasonable hypotheses as to why supervised exercise interventions produce more favorable outcomes compared to home-based exercise programs. In a supervised exercise class, the patient will engage in social interaction with other participants, which will improve mood, attitude and motivation which can be depressed in patients with a chronic illness such as ESKD requiring maintenance HD [56]. Especially for some elderly patients who may have become more socially isolated, this camaraderie can allow them to interact with others who share the same experiences in everyday life. During supervised exercise, patients will also have the advantage of receiving complete or semi-personalized attention in order to ensure that the intervention is being applied correctly in order to maximize results.

This finding is extremely relevant in today’s political environment. The Canadian provincial governments have decided to promote self-management as the main intervention for chronic disease in order to relieve some of the burden on the health care system. Using the Stanford program (USA) and the Expert Patient Programmed (UK) as models, the Ontario (Canada) government conducted a systematic review in 2008 in order to determine the efficacy of self-management strategies in chronic diseases [57]. The intervention involved patient education and counseling in order to inform patient about their disease and how to manage the symptoms. The aim is that patients will then take on the responsibility of managing their disease under self-supervision after the initial education. However, our results, at least in the chronic disease of ESKD on HD, show that relying on the patient as the sole supervisor of their own health and wellness program does not produce favorable results, as evidenced by ongoing deterioration of GS and distance walked. We believe that self-management in combination with periods of supervised intervention may be more beneficial when new deficits of gait or new challenges arise particularly in patients with ESKD/HD. Access to appropriate health professionals in a timely manner will help address the gait and mobility impairments, reduce the risk of falls and related consequences and in turn save valuable dollars associated with hospitalizations and need for institutional care. Further research is required to elucidate the benefits of supervised versus home-based exercise programs leading to clinically meaningful changes in GS and distances walked.

### Limitations

We have comprehensively reviewed the effect of chronic hemodialysis on physical function. We have not reviewed several factors that can impact the outcome of the studies. For example, the relative and absolute reliability of the outcome measures require consideration when used in studies utilizing repeated measures designs. We established that for a stable estimate of 6MWT, a minimum of two measures are required [58]. None of the studies included in this review collected the measure on two occasions for stable baseline value of the measure. We did not find any study that evaluated the relative and absolute reliability of the GS, or TUG in the population of interest.

The effect of whole-body fluid loss following HD may impact the assessment of the functional outcome measure collected. Hence, it is important to report the time of data collection in relation to the participants’ HD treatment schedule; e.g., data was collected on non-dialysis day, or just prior to HD treatment. Most of the studies included here did not categorically indicate the time of data collection.

It was not the intent of this review to comprehensively evaluate the effect of exercise/nutrition on gait parameters. We have reviewed the studies reporting the benefits of exercise /nutrition within the cluster of studies selected for addressing the primary objective of looking at the effect of HD on spatio-temporal gait parameters.

## 5. Conclusions

In conclusion, the results of this systematic review indicate that the people with ESKD on HD treatment experience deterioration of spatial and temporal characteristics of their gait. It is important to ensure that people with such conditions have access to a multi-disciplinary health-care team for timely intervention and to reduce the deleterious consequences of the disease and related disorders. This, in turn, may reduce the burden of kidney disease and related healthcare costs.

## Figures and Tables

**Table 1:** Characteristics of the studies included.

Study Name	Subgroup within Study	Outcome	Mean (Standard Deviation)	Sample Size (N)	Age of Participants in Years	Dialysis Vintage in Months (Mean (Standard Deviation) or Range)
Abe 2016 [22]	Participants on HD	Fast walking (m/s)	1.52 (0.34)	122	68.0 (9)	103.2 (103.2)
Blake 2004 [23]	Participants on HD	Normal walking (m/s)	1.31 (0.12)	12	42 (8.5)	Median 11.5 (4.25)
		Fast walking (m/s)	1.74 (0.18)	12		
Bulckaen 2011 [24]	Participants on HD (AWG group)	Treadmill walk Test	242 (208)	9	60 (9)	25.8
		6MWT (m)	436 (139)	9		
		Steps per day	2908 (1622)	9		
	Participants on HD (SWG group)	Treadmill walk Test	248 (197)	18	51 (12)	
		6MWT (m)	439 (124)	18		
		Steps per day	5000 (2904)	18		
Chang 2016 [25]	Participants on HD (intervention group)	6MWT (m)	389.9 (36.1)	21	54.2 15.2)	2.0 (1.3)
		Gait speed (m/s)	98.9 (23.9)	21		
	Participants on HD (control group)	6MWT (m)	387.2 (63.9)	25	54.6 12.7)	1.6 (0.9)
		Gait speed (m/s)	102.2 (17.6)	25		
Cheema 2007 [26]	Participants on HD (PRT group)	6MWT	496.6 (133.2)	24	60.0(15.3)	39.6 Mean Range (3.6–200.4)
	Participants on HD (Control group)	6MWT	406.4 (122.8)	25		
Cheema 2010 [27]	Participants on HD	6MWT (m)	450.6 (134.7)	49	62.5 (14.2)	26.4 (16.7)
		Normal walking (m/s)	1.0 (0.28)	49		
Golebiowski 2012 [28]	Participants on HD	6MWT (m)	0.99(1.3)	29	64.2 (13.1)	4–192 Range
Headley 2002 [29]	Participants on HD	6MWT (m)	522.1 (46.2)	10	42.8 (4.4)	41.6 (19)
		Fast walking (m/s)	1.83 (0.13)	10		
		Normal walking (m/s)	1.21 (0.61)	10		
Henrique 2010 [30]	Participants on HD	6MWT (m)	509 (91.9)	14	47.6 (12.7)	93.7 (43.9)
Hotta 2015 [31]	Participants on HD with sarcopenia	Gait speed (m/s)	1.4 (0.1)	14	70.5 (2.2	53.3 (5.5)
	Participants on HD without sarcopenia	Gait speed (m/s)	2.0 (0.1)	19	65.5 (2.4)	50.1 (8.8)
Jeong 2015 [32]	Patients with LVDD	Gait speed (m/s)	0.7 (0.2)	40	54.5 (11.1)	42.9 (37.9)
		ISWT (seconds)	188 (102.1)	40		
	Patients without LVDD	Gait speed (m/s)	0.9 (0.3)	42		
		ISWT (seconds)	261.4 (117.7)	42		
Jin 2017 [33]	Participants on HD with T2DM	Gait speed (m/s)	0.87 (0.21)	35	63.89 (9.57)	54.26 (49.61)
	Participants on HD without T2DM	Gait speed (m/s)	1.0 (0.23)	25	56.4 (14.67)	81.64 (70.12)
Johansen 2001 [34]	Participants on HD	Gait speed (m/s)	1.13 (0.34)	46	52 (17)	27.6 (27.5)
Johansen 2001 [35]	Participants on HD	Gait Speed (m/s)	1.15 (0.34)	39	52 (16)	28.8 (28.8)
Johansen 2003 [5]	Participants on HD	Gait speed (m/s)	1.0 (0.33)	38	55 (15)	34.8 (32.7)
Johansen 2015 [36]	Participants on HD	Gait speed (m/s)	0.93 (0.68)	68	69 (14)	44.4 Mean Range (19.2–75.6)
Johansen 2014 [37]	Participants on HD (Non-frail group)	Normal walking speed m/s	1.0 (0.2)	321	53.8 (14.4)	44.4 Mean Range (19.2–75.6)
Koh 2010 [38]	HD participants on home exercises	6MWT (m)	444 (127)	14	52.1 (13.6)	37.0 (31.1)
		TUG in seconds	5.7 (2.0)	14		
	HD participants on intradialytic exercise	6MWT (m)	463 (127)	14	52.3 (10.9)	32.1 (26.7)
		TUG in seconds	5.8 (1.5)	14		
	HD participants receiving usual care	6MWT (m)	431 (160)	16	51.3 (14.4)	25.8 (22.2)
		TUG in seconds	6.3 (2.5)	16		
Kutsuna 2010 [39]	Participants on HD	Fast walking (m/s)	1.52 (0.42)	153	64 (11)	93.6 (80.4)
		Normal walking (m/s)	1.13 (0.28)	153		
Lane 2013 [40]	Participants on HD	SWT (in seconds)	251 (120)	42	44(5)	51 (42)
Malagoni 2008 [41]	Participants on HD (control group)	6MWT (m)	275 (69)	7	66 (14)	90 (73)
	Participants on HD (experimental group)	6MWT (m)	308 (105)	13	62 (10)	78 (50)
Manfredini 2007 [42]	Participants on HD	6MWT (m)	283 (122)	16	65.1 (11.4)	82 (77)
		Max. treadmill speed m/s	0.92 (0.31)	16		
Mercer 2002 [43]	Participants on HD + PD (experimental group)	50 m walk test	146 (38.1)	7	63..0 (14.5)	30 (18)
	Participants on HD + PD (control group)	50 m walk test	139.1 (21.3)	9	59 (12.3)	45.6 (33.6)
Painter 2000 [44]	Participants on HD	6MWT (m)	347.10 (127.10)	44	55.9 (15.15)	33.7 (35.6)
		Fast walking (m/s)	1.30 (0.40)	131		
		Normal walking (m/s)	0.9 (0.26)	131		
Shin 2013 [45]	Participants on HD	Normal walking (m/s)	1.0 (0.247)	14	50.0 (11.8)	51.6 (35.5)
		Cadence (steps/min)	100.1 (12.6)	14		
		Step length (cm)	59.7 (12.7)	14		
		Step width (cm)	62.05 (11.7)	14		
		Double support (%GC)	34.5 (6.8)	14		
		Swing phase (%GC)	32.9 (3.5)	14		
Tao 2015 [46]	Participants on HD (experimental group)	Fast walking (m/s)	1.17 (0.27)	56	53.02 (11.62)	83.46 (63.7)
		Normal walking (m/s)	1.21(0.25)	57		
	Participants on HD (Control group)	Fast walking (m/s)	1.6 (0.4)	56	56.68 (9.67)	84.7 (70.55)
		Normal walking (m/s)	1.17 (0.27)	56		
Tomayko 2015 [47]	Participants on HD receiving Nutrition (Whey) group	Gait speed (m/s)	0.84 (0.27)	11	57 (4.8)	Not reported
		Shuttle walk test	216 (36)			
		TUG in seconds	7.3 (1.0)			
	Participants on HD (Control group)	Gait speed (m/s)	0.86 (0.29)	15	53.3 (2.4)	
		Shuttle walk test	222 (34)			
		TUG in seconds	7.7 (0.9)			
	Participants on HD receiving Nutrition (Soy) group	Shuttle walk Test	268 (35)	12	52.5 (4.3)	
		TUG in seconds	7.6 (1.1)			
		Gait speed (m/s)	0.86 (0.23)			

6MWT: 6 min walk test; AWG: advised walking group; cm: centimeters; HD: hemodialysis, m: meters, m/s: meters per second; GC: gait cycle; ISWT: intermittent shuttle walk test; LVDD: left ventricular diastolic dysfunction; m: meters, m/s: meters per second; Max: maximum; PD: peritoneal dialysis; SWG: supervised walking group; SWT: shuttle walk test; T2DM: type II diabetes mellitus; TUG: timed get up and go test.

**Table 2:** Effect of hemodialysis on gait speed.

Study Name	Subgroup within Study	Outcome Measure	Mean	95% Confidence Interval	
				Lower Limit	Upper Limit
Blake 2004 [23]	Participants on HD	Normal walking (m/s)	1.31	1.24	1.38
Chang 2016 [25]	Participants on HD (intervention group)	Gait Speed (m/s)	1.02	0.95	1.09
	Participants on HD (control group)	Gait Speed (m/s)	0.99	0.89	1.09
Cheema 2010 [27]	Participants on HD	Normal walking (m/s)	1.00	0.92	1.08
Headley 2002 [29]	Participants on HD	Normal walking (m/s)	1.21	0.83	1.59
Jeong 2015 [32]	Patients with LVDD	Gait Speed (m/s)	0.70	0.64	0.76
	Patients without LVDD	Gait Speed (m/s)	0.90	0.81	0.99
Jin 2017 [33]	Participants on HD with T2DM	Gait Speed (m/s)	0.87	0.80	0.94
	Participants on HD without T2DM	Gait Speed (m/s)	1.00	0.91	1.09
Johansen 2001 [34]	Participants on HD	Gait Speed (m/s)	1.13	1.03	1.23
Johansen 2001 [35]	Participants on HD	Gait Speed (m/s)	1.15	1.04	1.26
Johansen 2003 [5]	Participants on HD	Gait Speed (m/s)	1.00	0.90	1.11
Johansen 2015 [36]	Participants on HD	Gait Speed (m/s)	0.93	0.77	1.09
Johansen 2014 [37]	Participants on HD (Non-frail group)	Normal walking (m/s)	1.00	0.98	1.02
Kutsuna 2010 [39]	Participants on HD	Normal walking (m/s)	1.13	1.09	1.17
Painter 2000 [44]	Participants on HD	Normal walking (m/s)	0.90	0.86	0.94
Shin 2013 [45]	Participants on HD	Normal walking (m/s)	1.00	0.87	1.13
Tao 2015 [46]	Participants on HD (Control group)	Normal walking (m/s)	1.17	1.10	1.24
	Participants on HD (experimental group)	Normal walking (m/s)	1.21	1.15	1.27
Tomayko 2015 [47]	Participants on HD receiving Nutrition (Whey) group	Gait Speed (m/s)	0.84	0.68	1.00
	Participants on HD (Control group)	Gait Speed (m/s)	0.86	0.71	1.01
	Participants on HD receiving Nutrition (Soy) group	Gait Speed (m/s)	0.86	0.73	0.99
			1.01	0.95	1.07

HD: hemodialysis, m: meters, m/s: meters per second; LVDD: left ventricular diastolic dysfunction; m/s: meters per second; T2DM: type II diabetes mellitus.

**Table 3:** Effect of hemodialysis on fast walking speed.

Study Name	Subgroup within Study	Outcome	Statistics for Each Study	95% Confidence Interval	
			Mean	Lower Limit	Upper Limit
Abe 2016 [22]	Participants on HD	Fast walking (m/s)	1.52	1.46	1.58
Blake 2004 [23]	Participants on HD	Fast walking (m/s)	1.74	1.64	1.84
Headley 2002 [29]	Participants on HD (HEP)	Fast walking (m/s)	1.83	1.75	1.91
Kutsuna 2010 [39]	Participants on HD	Fast walking (m/s)	1.52	1.45	1.59
Manfredini 2007 [42]	Participants on HD	Max. Treadmill Speed (m/s)	0.92	0.77	1.07
Painter 2000 [44]	Participants on HD	Fast walking (m/s)	1.30	1.23	1.37
Tao 2015 [46]	Participants on HD (control group)	Fast walking (m/s)	1.60	1.50	1.70
	Participants on HD (experimental group)	Fast walking (m/s)	1.17	1.10	1.24
	Random effects		1.45	1.28	1.62

HD: hemodialysis; HEP: home exercise program; Max: maximum; m/s: meters per second.

**Table 4:** Effect of hemodialysis on timed get up and go test.

Study Name	Subgroup within Study	Statistics for Each Study	95% Confidence Limit	
		Mean (Seconds)	Lower Limit	Upper Limit
Koh 2010 [38]	Participants on HD (home exercises	5.70	4.65	6.75
	Participants on HD (intradialytic exercises)	5.80	5.01	6.59
	Participants on HD (usual care)	6.30	5.08	7.52
Tomayko 2015 [47]	Participants on HD (nutrition (Whey) group)	7.30	6.71	7.89
	Participants on HD (control group)	7.70	7.24	8.16
	Participants on HD (nutrition (Soy) group)	7.60	6.98	8.22
	Random effects	6.82	6.13	7.52

HD: hemodialysis.

**Table 5:** Effect of hemodialysis on six minute walk test.

Study name	Subgroup within Study	Statistics for Each Study	95% Confidence Limits	
		Mean	Lower Limit	Upper Limit
Bulckaen 2011 [24]	Participants on HD AWG	436.00	345.19	526.81
	Participants on HD SWG	439.00	381.72	496.28
Chang 2016 [25]	Participants on HD (control group)	387.20	362.15	412.25
	Participants on HD (intervention group)	389.90	374.46	405.34
Cheema 2007 [26]	Participants on HD (control group)	406.42	358.25	454.59
	Participants on HD (experimental group)	496.61	443.29	549.93
Cheema 2010 [27]	Participants on HD (experimental group)	450.60	412.88	488.32
Headley 2002 [29]	Participants on HD	522.10	493.47	550.73
Henrique 2010 [30]	Participants on HD	509.00	460.86	557.14
Koh 2010 [38]	Participants on HD (home exercises)	444.00	377.47	510.53
	Participants on HD (intradialytic exercises)	463.00	396.47	529.53
	Participants on HD (usual care)	431.00	352.60	509.40
Malagoni 2008 [41]	Participants on HD (control group)	275.00	223.89	326.11
	Participants on HD (experimental group)	308.00	250.92	365.08
Manfredini 2007 [42]	Participants on HD	283.00	223.22	342.78
Painter 2000 [44]	Participants on HD	347.10	309.55	384.65
	Random effects	411.56	377.02	446.10

AWG: advised walking group; HD: hemodialysis; SWG: supervised walking group.

**Table 6:** Effect of hemodialysis on walking time.

Study Name	Subgroup within Study	Outcome	Statistics for Each Study	95% Confidence Interval	
			Mean Time in Seconds	Lower Limit	Upper Limit
Bulckaen 2011 [24]	Participants on HD (AWG)	Treadmill walk test	242.00	106.11	377.89
	Participants on HD (SWG)	Treadmill walk test	248.00	156.99	339.01
Jeong 2015 [32]	Patients with LVDD	ISWT (seconds)	188.00	156.36	219.64
	Patients without LVDD	ISWT (seconds)	261.40	225.80	297.00
Lane 2013 [40]	Participants on HD	SWT (in seconds)	25.10	21.47	28.73
Mercer 2002 [43]	Participants on HD + PD (control group)	50 m walk test	139.10	125.18	153.02
	Participants on HD + PD (experimental group)	50 m walk test	146.00	117.78	174.22
Tomayko 2015 [47]	Participants on HD Nutrition (Whey) group	Shuttle walk test	216.00	194.73	237.27
	Participants on HD control group	Shuttle walk test	222.00	204.79	239.21
	Participants on HD Nutrition (Soy) group	Shuttle walk test	268.00	248.20	287.80
	Random		193.55	116.36	270.73

AWG: advised walking group; HD: hemodialysis; ISWT: intermittent shuttle walk test; LVDD: left ventricular diastolic dysfunction; PD: peritoneal dialysis. SWG: supervised walking group.

**Table 7:** Results of comparison of gait speed between participants on hemodialysis and age-matched participants with no kidney disease.

Study Name	Outcome	Statistics for Each Study	95% Confidence Limit		*p*-Value
		Hedges’s g	Lower Limit	Upper Limit	
Blake 2004 [23]	Normal walking (m/s)	1.58	0.69	2.47	0.00
Johansen 2003 [5]	Gait Speed (m/s)	1.45	0.85	2.06	0.00
Johansen 2001 [34]	Gait Speed (m/s)	0.62	0.02	1.22	0.04
Shin 2013 [45]	Normal walking (m/s)	1.60	0.77	2.44	0.00
	Random effects	1.26	0.76	1.76	0.00

**Table 8:** Gait parameters using GAITRite Mat.

Study Name	Outcome	Hedges’s g	Std Err
Shin 2013 [45]	Cadence (steps/minute)	1.03	0.39
	Double Support (%GC)	−1.03	0.39
	Step length (cm)	1.21	0.40
	Step width (cm)	1.18	0.40
	Swing Phase (%GC)	0.98	0.39

cm: centimeters; GC: gait cycle.
